# Human activities and landscape features interact to closely define the distribution and dispersal of an urban commensal

**DOI:** 10.1111/eva.12650

**Published:** 2018-07-21

**Authors:** Qian Tang, Gabriel Weijie Low, Jia Ying Lim, Chyi Yin Gwee, Frank E. Rheindt

**Affiliations:** ^1^ Department of Biological Sciences National University of Singapore Singapore

**Keywords:** hierarchical distance sampling, invasive species, landscape genomics, pigeon, urban environment

## Abstract

The rock pigeon, *Columba livia*, is a cosmopolitan human commensal, domesticated thousands of years ago. However, the human‐mediated factors governing its distribution and dispersal are not well understood. In this study, we performed (a) hierarchical distance sampling on ~400 island‐wide point transects, (b) a population genomic inquiry based on ~7,000 SNPs from almost 150 individuals, and (c) landscape genomic analyses on the basis of extensive ecological and socio‐economic databases to characterize the distribution and dispersal patterns of rock pigeons across Singapore. Our distance sampling results indicated that the volume of intentional “mercy feeding” and availability of high‐rise buildings are the most reliable predictors of high pigeon densities in Singapore. Genomic analyses demonstrated that rock pigeons in Singapore form a single population possibly derived from rapid expansion from a genetically homogenous group of founder individuals. In specific, rock pigeons in Singapore lack sex‐biased dispersal and are clustered with a genetic patch size of ~3 km. Landscape genomic analyses of great precision pointed to the presence of dense trees as agents of resistance to dispersal, whereas a high road density reduces this resistance. By pinpointing a range of ecological and socio‐economic variables determining the distribution and dispersal of pigeons, our study provides urban planners with the tools for optimal management of this human commensal, such as a curtailment of the practice of mercy feeding and modifications to the urban landscape to reduce pigeon density and to lower the likelihood of repopulation by dispersal.

## 1 INTRODUCTION

Rapid urbanization in recent decades has created a unique artificial landscape, the urban landscape, which now occupies a significant proportion of the earth's surface (Seto, Fragkias, Güneralp, & Reilly, 2011). Many recent studies have explicitly evaluated the impact of urbanization on the evolution of urban dwelling organisms with a purpose of conserving native species (Beninde et al., 2016; Johnson & Munshi‐South, 2017; Munshi‐South, Zolnik, & Harris, 2016), whereas relatively fewer such studies (Richardson et al., 2017) have explicitly assessed such an impact on the evolution of human commensal species. Given that human commensal species are considered socio‐economically more important than noncommensal species because of a larger habitat overlap with humans, the evolutionary consequences of human‐driven ecological processes on human commensal species remain understudied (Hulme‐Beaman, Dobney, Cucchi, & Searle, 2016). Understanding the human impact on the distribution and dispersal of human commensal species may help us adjust our policies and urban design to alter human‐induced eco‐evolutionary changes and ultimately optimize ecological and societal consequences (Hendry, Gotanda, & Svensson, 2017).

The rock pigeon, *Columba livia* (Gmelin), is one of the oldest and most common cosmopolitan human commensal species (Johnston & Janiga, 1995). Domesticated in the early stages of human civilization, the rock pigeon has been bred into a large number of varieties under artificial selection (Shapiro & Domyan, 2013) and probably constitutes one of the most suitable organisms to study the impact of artificial selection on evolution (Darwin, 1859). Urban rock pigeons are now found in most cities globally and are the feral descendants of domesticated rock pigeons (Shapiro & Domyan, 2013; Stringham et al., 2012).

On the one hand, as an integral part of urban biodiversity, rock pigeons occupy a unique niche and may provide important services to urban ecosystem, for example, by serving as the prey of native raptors (Kenward, 1996). On the other hand, burgeoning populations of rock pigeons in many cities incur costs to public health (Haag‐Wackernagel & Bircher, 2010) and damage to urban infrastructure (Haag‐Wackernagel & Geigenfeind, 2008). To mitigate rock pigeons’ impacts on urban ecology and human society, that is, to reduce their potential costs to human well‐being while maintaining their role in urban ecosystem services, we need to understand how human activities influence their distribution and dispersal, and hence their ecology and evolution.

Many previous studies have attempted to evaluate human influences on rock pigeons’ distribution and dispersal in urban landscapes. However, previous studies using traditional observation and distance sampling methods were not always able to accurately connect abundance with environmental covariates and human activities, rendering interpretations of results largely dependent on the local context (e.g., Hetmański, Bocheński, Tryjanowski, & Skórka, 2010; Przybylska et al., 2012; Sacchi, Gentilli, Razzetti, & Barbieri, 2002). Traditional telemetric and GPS tracking has successfully revealed patterns of mobility but often without paying heed to concepts of dispersal (e.g., Rose, Nagel, & Haag‐Wackernagel, 2006). Two population genetic studies have shed light on dispersal patterns of the Paris population: Jacob, Prévot and Baudry (2015) indicated a rock pigeon dispersal behavior that is independent of geographical distance (when <20 km) across Paris. Afterward, Jacob, Prévot and Baudry (2016) pointed to widespread inbreeding and preference of genetically similar mates among rock pigeons in Paris. However, neither study explicitly explored the association between genetic patterns and socio‐economic factors.

To investigate the rock pigeon's distribution, dispersal, and associated human‐mediated determinants across urban settings, we use a large dataset from across the city of Singapore combining hierarchical distance sampling (Royle, Dawson, & Bates, 2004) with population and landscape genomic approaches. For the hierarchical distance sampling, we employ environmental covariates commonly used in urban ecological studies (LaPoint et al., 2015). For the population genomic component, we adopt the concept of individual‐based population genetics (LaPoint et al., 2015) in both sampling scheme design (Landguth & Schwartz, 2014) and spatially explicit analysis (Keis et al., 2013). Using rock pigeons from across Singapore, we aim to discover how certain human‐mediated determinants can closely define the distribution and dispersal of human commensal species and to shed light on how modifying human influences can optimize ecological and societal outcomes.

## 2 MATERIAL AND METHODS

### 2.1 Study area

The study was conducted on the main island of Singapore (1°22′N, 103°48′E), situated at the southern tip of the Malay Peninsula. With a total land area of 719.1 km^2^, Singapore is densely populated with an estimated 5.61 million people. Singapore has a tropical rainforest climate with limited fluctuations in temperature (mean daily temperatures range from 23.9°C to 32.3°C), rainfall (mean monthly rainfall ranges from 112.8 mm to 318.6 mm), and humidity (mean daily humidity ranges from 64.5% to 95.5%) throughout the year (Singapore Department of Statistics, 2017). Most of the human population in Singapore resides in the eastern half of the island. Central Singapore is a large forest reserve with a few freshwater reservoirs; the northwest is largely occupied by forested areas for military use; and the southwest is dominated by port and industrial zones (Supporting Information Figure S1b). Rock pigeons in Singapore are considered feral as there is no pigeon racing industry in Singapore and live pigeons are rarely imported. All the research protocols in this study were reviewed and permitted by the Agri‐Food and Veterinary Authority (AVA) of Singapore. Protocols associated with animal genetic sampling were approved by the Institutional Biosafety Committee (IBC, Reference Number: 2016‐00192A) and the National University of Singapore Institutional Animal Care and Use Committee (IACUC, Protocol Number: B16‐0192).

### 2.2 Distance sampling and density estimation

**Figure 1:**
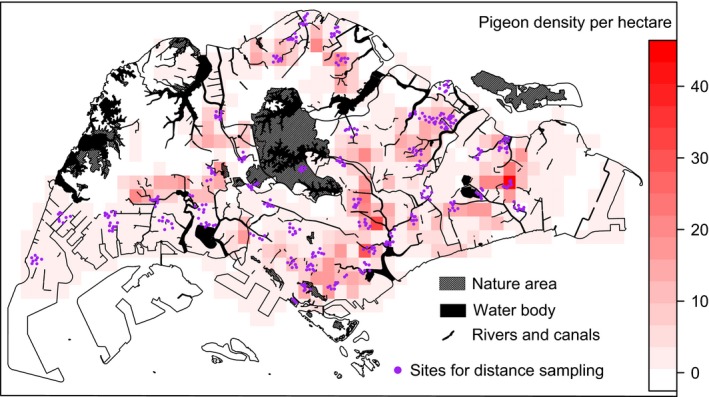
Predicted density (number of individuals per hectare) of rock pigeons across Singapore. Darker red cells are areas that are predicted to have a higher density of pigeons. Purple circles are distance sampling sites

We carried out point count distance sampling to obtain an unbiased estimate of rock pigeon abundance across the main island of Singapore. Surveys were conducted at 400 sampling sites (Figure 1) with single visits between 7:00 a.m. and 11:30 a.m. from June 2016 to January 2017. The duration of surveys at each site was 10 min, during which the surveyor measured the detection distance to pigeons at the point where they were first sighted using a Forestry PRO Laser Rangefinder (Nikon, Japan). Rock pigeons within a 2 m diameter of one another were considered a cluster (Ronconi & Burger, 2009). When dealing with clusters, we measured the distance to the center of the cluster and recorded the numbers of individuals.

In addition, we collected four environmental covariates: intensity of human “mercy feeding” (based on ~7,000 incidents reported to government agencies across 3 years [2015‐2017]), that is, humans deliberately providing pigeons with bread, rice, or similar food (F); landscape (L); resident human population density (P); and road density (R). We obtained landscape information via Google Earth satellite imagery (image date: December 11, 2016). Adapting the “local climate zone” classification scheme (Stewart & Oke, 2012) to Singapore's context, we subcategorized the urban landscape in Singapore under six categories: high‐rise, low‐rise, industrial zones, open vegetation, dense trees, and bare ground (details see Table 1). We sampled across all landscapes such that they approximated the actual proportions of each landscape category across Singapore. However, due to accessibility constraints, we surveyed relatively fewer sites in the dense tree landscape and none of the sites in the bare ground landscape (most of which constituted out‐of‐bounds construction sites). All other environmental covariates were collected from a Singaporean government open source dataset or associated government agencies. We rasterized all of Singapore into 1,250 cells (25 cells North–South wise, 50 cells East–West wise, each cell covering an area ~1 km^2^), each cell characterized by four environmental covariates (Supporting Information Figure S1).

**Table 1:** Categories of urban landscape used in this study

Landscape types	Descriptions	Examples
High‐rise (HR)	High‐rise buildings (>7 stories) densely arranged on paved land cover with scattered trees/shrubs.	Public housing estates, high‐rise condominiums, and commercial buildings.
Low‐rise (LR)	Low‐rise buildings (1–7 stories) densely arranged on paved land cover with scattered trees/shrubs.	Private residential estates, shop houses, schools.
Industrial zones (ID)	Industrial buildings (1–7 stories) and structures densely arranged on paved land cover, with few or the absence of trees.	Factories, stacks, containers, heavy machinery.
Open vegetation (OV)	Grass or herbaceous plants/crops forming pervious land cover, with few or scattered trees.	Parks, golf courses, recently cleared woodlands.
Dense trees (DT)	Densely wooded landscape of trees, with pervious land cover.	Forests, abandoned plantations, wastelands.
Bare ground (BG)	Bare sand, bare soil, or paved land cover with no plants.	Airbase, recently reclaimed land, construction sites.

**Figure 2:**
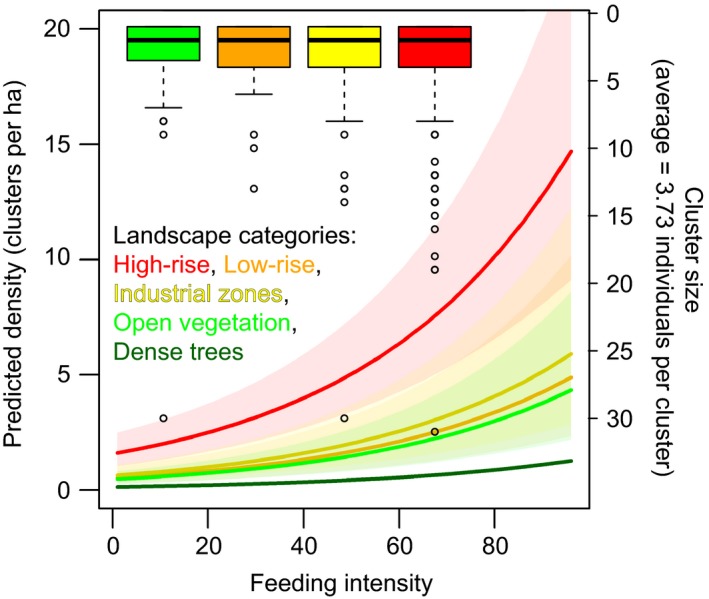
Correlation between feeding intensity and expected rock pigeon density in different categories of landscape according to the L‐FL model. Cluster sizes are not significantly different among different landscape categories (shown as box plots in the top of the figure). As there was no sighting of pigeons during distance sampling in the dense tree landscape, box plots of cluster size and confidence intervals of the L‐FL model for the dense tree landscape are not available

We estimated pigeon density with hierarchical distance sampling modeling (Royle et al., 2004), using the function *distsamp* as implemented in the R package *unmarked* (Fiske & Chandler, 2011). An observation of either an individual or a cluster of individuals was input as one count. As cluster sizes were not significantly different across landscapes (Figure 2), we calculated the average cluster size which was then multiplied with our predicted density in the final pigeon density estimate. We tested all four detection functions in *unmarked* and decided to use “*hazard‐rate*” because it demonstrated better predictions than the other three functions across preliminary models. We used different combinations of environmental covariates to model both detection probability (*p*) and abundance (λ). By comparing the Akaike information criterion (AIC) values of all models (Table S1), we selected the best model, the L‐FL model, which uses landscape for *p* estimation and both landscape and feeding intensity for λ estimation. Using the L‐FL model, we calculated the predicted density of pigeons for all 1,250 (25 × 50) cells according to the landscape and mercy feeding intensity in each cell. Due to limited data availability, we were unable to calculate standard error (*SE*) in the dense tree landscape or model the density in the bare ground landscape. However, this was unlikely to compromise our estimate of rock pigeon density across Singapore as the density estimate for the dense tree landscape was approximately zero and the bare ground landscape only comprised a relatively small proportion (3.28%, 41 of 1,250 cells) of the entire area of Singapore. Even if rock pigeons do indeed inhabit the bare ground landscape, final density should only be slightly underestimated.

### 2.3 Genetic sample collection and wet lab protocol

**Figure 3:**
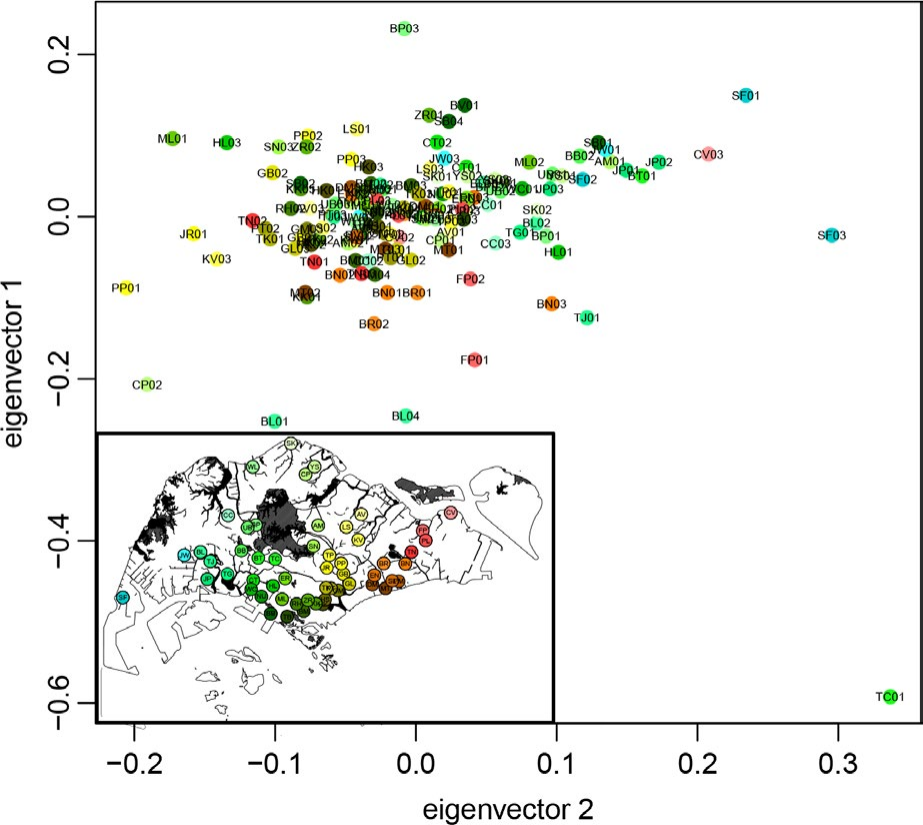
Principal component analysis of rock pigeons across Singapore. Map in the left bottom indicates sampling locations. The color scheme indicates the geographical cline of the sample distribution

A total of 144 rock pigeon blood/tissue samples were collected from 55 locations across Singapore (Figure 3), 129 of which were captured live using box‐stick traps and the remaining 15 were carcasses from freshly dead individuals provided by the public. Overall, our sampling was evenly distributed across urban Singapore (Figure 3) and each sampling site was kept at least 500 m apart from one another. Blood was drawn from each live individual via brachial venipuncture, then transferred into 2.5 ml Eppendorf tubes, and topped up with absolute ethanol. For each carcass, 2 mm^3^ of breast muscle was extracted, minced, and preserved in absolute ethanol in a 2.5 ml Eppendorf tube. All blood and tissue samples were stored in a −20°C freezer for subsequent processing.

We extracted genomic DNA using the DNeasy Blood & Tissue Kit (Qiagen, Germany) following the protocol supplied by the manufacturer. For double‐digest restriction enzyme‐associated sequencing library preparation, we followed the protocol used by Low et al. (2018). Prepared libraries were pooled and submitted for Next‐Generation Sequencing (Illumina HiSeq 4500). In addition, we identified the sex of each pigeon sample by amplifying the chromo‐helicase‐DNA‐binding protein genes (CHD1) on pigeons’ Z (CHD1Z) and W (CHD1W) chromosomes using the 2500F/2718R primer set (Fridolfsson & Ellegren, 1999). We performed the PCRs using DreamTaq (Thermo Fisher Scientific, USA) following a protocol by Vucicevic et al. (2013) with a reduction to five elongation cycles. PCR products were visualized with gel electrophoresis. Based on the length of the PCR products (400 kb for CHD1W, 600 kb for CHD1Z), we successfully identified the sex of 139 individuals. The sex of the remaining five individuals was labeled as “unknown” because CHD1 gene amplification was unsuccessful.

### 2.4 Bioinformatic protocol

Based on the results from sequence quality checks using *FastQC* (Babraham Bioinformatics, USA), we decided to retain all sequences without further truncation. After demultiplexing and clean‐up using the command *process_radtag* implemented in *Stacks v1.3* (Catchen, Amores, Hohenlohe, Cresko, & Postlethwait, 2011; Catchen, Hohenlohe, Bassham, Amores, & Cresko, 2013), 456 million reads (average 3.16 million per individual [454,855–8,568,180 reads]) of ~400 bp length each were retained. We then aligned the reads to the complete rock pigeon genome (Shapiro et al., 2013) using BWA‐MEM as implemented in *BWA v.0.7.15* (Li, 2013). We input the alignments into *Stacks* to call single nucleotide polymorphisms (SNPs) using the command *ref_map.pl*, in which we regarded all individuals as a single population. All the SNPs called were filtered for linkage disequilibrium using *PLINK v1.9* (Chang et al., 2015; Purcell et al., 2007), and SNPs containing missing data were removed, retaining 7,013 SNPs (pairwise linkage disequilibrium lower than 0.9). We explored additional SNP calling regimes allowing for 1% and 5% missing data and trialed them by computing summary statistics, pairwise relatedness, and performing principal component analysis (see below). However, given that results were not qualitatively different, we exclusively report on the results of the principal SNP set with no missing data.

### 2.5 Population genomic analytical approaches

To examine the genomic attributes of rock pigeons in Singapore as an entire population, we calculated expected heterozygosity (*H*_e_), observed heterozygosity (*H*_o_), and inbreeding coefficient (*F*_IS_) using *GenAlEx v6.503* (Peakall & Smouse, 2012). We estimated pairwise relatedness (r) across all individual pairs using a maximum likelihood algorithm (Choi, Wijsman, & Weir, 2009; Milligan, 2003) as implemented in the R package *SNPRelate* (Zheng et al., 2012). Four pairs (eight individuals) were detected to be closely related (r > 10%), constituting potential kin. As closely related individuals may compromise subsequent principal component analysis (PCA), one individual from each related pair was removed. We used PCA as implemented in *SNPRelate* to detect potential population subdivision among 140 individuals after excluding the closely related individuals. For all other analyses, the full dataset of 144 individuals was used.

To detect population subdivision of rock pigeons across Singapore, we performed maximum likelihood estimation of individual ancestries using *ADMIXTURE v 1.3* (Alexander, Novembre, & Lange, 2009). In the ADMIXTURE analysis, multiple proposed numbers of subdivisions (*k *=* *2–10, 15, 20, 25, 30, 35, 40, 45, 50) were tested. The most likely value of *k* was determined by calculation of cross‐validation errors. Parallel to *ADMIXTURE*, we performed Bayesian inference of possible population subdivision using the software *STRUCTURE v2.3.4* (Pritchard, Stephens, & Donnelly, 2000). We tested numbers of population subdivisions (*K*) ranging from one to ten using 10 iterations each with a burnin of 100,000 generations followed by 500,000 generations of Markov Chain Monte Carlo (MCMC) iterations. We used *Delta K* (Evanno, Regnaut, & Goudet, 2005) as implemented in *StructureHarvester Web v0.6.94* (Earl, 2012) on the *STRUCTURE* results to determine the most likely *K* value of population subdivision. We downloaded the output files of the best *K* and inputted them into *Clumpp v1.1.2* (Jakobsson & Rosenberg, 2007) to integrate and summarize iterations.

We inferred the demographic history of rock pigeons in Singapore with approximate Bayesian computation (ABC) in DIYABC v2.1 (Cornuet et al., 2014). We followed Low et al. (2018) to simulate three possible scenarios (population expansion, population recovery, and population contraction) with all associated single‐population summary statistics. We employed a minimum allele frequency of 0.01, resulting in a subset of 4,049 SNPs for the actual coalescent simulations. We performed a polychotomous logistic regression on the summary statistics of each simulated model to estimate their posterior probability and then estimated the posterior predictive error rate for the most likely scenario, before further estimating relevant demographic parameters. These were the ancestral, bottleneck, and present‐day effective population sizes (*N*_*anc*_, *N*_*bot*_, *N*_*rec,*_ respectively) and the time of bottleneck and recovery (*t*_*bot*_ and *t*_*rec,*_ respectively). At last, we estimated the bias and error rates of these parameter estimates.

Alone, we estimated present‐day effective population size (*N*_*e*_) using the molecular coancestry approach (Nomura, 2008) implemented in NeEstimator v2.1 (Do et al., 2014).

We calculated a pairwise genetic distance matrix using *GenAlEx* while enforcing the Codon‐Genotypic option (Peakall, Smouse, & Huff, 1995; Smouse & Peakall, 1999). To gauge the spatial dispersal pattern of rock pigeons across Singapore, we performed spatial autocorrelation (Smouse & Peakall, 1999; Smouse, Peakall, & Gonzales, 2008) to determine the most likely breeding range and genetic patch size. To detect sex‐biased dispersal (Banks & Peakall, 2012), we performed separate spatial autocorrelations with three datasets—among male individuals, among female individuals, and among all individuals sampled. For all three analyses, we set 15 even distance classes with 2 km as the interval. All analyses were performed with 999 permutations to simulate the null model of nonspatial correlation, and 999 bootstraps and tests of heterogeneity to validate the statistical significance of the observed data.

We examined the relationship between geographical influence and dispersal of rock pigeons in Singapore using the spatially explicit and individual‐based DResD approach (Keis et al., 2013), which calculates the geographical distribution of isolation‐by‐distance (IBD) residuals to indicate possible barriers and corridors for dispersal. Each IBD residual is a deviation of a pairwise comparison from the IBD trend. Positive IBD residuals indicate a relatively large genetic dissimilarity between two individuals across a relatively small geographical distance; negative IBD residuals indicate a relatively small genetic dissimilarity across a relatively large geographical distance. Positive IBD residuals accumulate at sites characterized by a lack of dispersal while negative IBD residuals accumulate at sites with high dispersal. We used the pairwise genetic distance matrix calculated in *GenAlEx* as the input. To calculate the statistical significance and power of DResD results, we ran 1,000 randomized iterations and 250 bootstraps. We then calculated and visualized the correlation between resistance of dispersal and different environmental covariates using R packages *PerformanceAnalytics* (Carl, Peterson, Boudt, & Zivot, 2009) and *corrplot* (Wei & Simko, 2013). Then, two matrices were generated, one using overall residuals and the other using statistically significant residuals.

## 3 RESULTS

### 3.1 Population density

Our analysis suggested that the best model (L‐FL) to predict population density of rock pigeons in Singapore accounted for two environmental covariates: mercy feeding intensity and landscape (Table S1). According to the model, landscape was associated with detection probability (*p*), whereas landscape together with mercy feeding intensity was associated with abundance (λ). This model (λ = *e* ^[0.437 + 0.023 × *F* + *L*]^, where *F* is the feeding intensity; *L* is the coefficient of each type of landscape [high‐rise 2.544, low‐rise 1.421, industrial zones 1.586, open vegetation 1.255]) showed that pigeon density increased more dramatically with increasing mercy feeding intensity in a high‐rise landscape compared to other landscapes (Figure 2). The model predicted no significant differences in low‐rise, industrial zones, and open vegetation landscapes at equal feeding intensity.

Multiplying the average cluster size of 3.73 individuals with the predicted density (cluster per hectare), we derived an average rock pigeon density in Singapore at 3.59 ± 1.14 individuals per hectare. The density in different landscapes varied: high‐rise (12.1 ± 2.48), low‐rise (2.48 ± 0.82), industrial zones (2.34 ± 0.66), open vegetation (1.69 ± 0.48), and dense trees (0.47). Given the area of the main island of Singapore (excluding the bare ground landscape) of 52,909 hectares, the total estimate of rock pigeons across Singapore amounts to 189,943 (129,627–250,259) individuals. In general, rock pigeon density was higher in the east than in the west of Singapore (Figure 1).

### 3.2 Population structure

Pairwise relatedness (r) ranged from 0 to 23.98%, with an average of 0.27%. All eight individual pairs (16 individuals) with r > 5% were collected at the same sites. The PCA result revealed that all individuals formed a single population in which geographically adjacent individuals are not necessarily genetically similar (Figure 3). Maximum likelihood clustering using *ADMIXTURE* inferred a most likely scenario of two population subdivisions of rock pigeons in Singapore (Supporting Information Figure S2). However, these two inferred population subdivisions had no geographical basis; individuals containing genetic components of both inferred populations are common across the whole of Singapore. Our Bayesian inference clustering with *STRUCTURE* also displayed a homogeneous pattern. The most likely clustering was at *K *=* *3, where all individuals (with an average of 91.9% and a minimum of 88.77% of their genomic composition) belong to a single genetic cluster with minor genetic components from the other two.

### 3.3 Historical and present demographic status

ABC results support the population recovery scenario as most likely (logistic posterior probability = 0.573 [0.561, 0.585; 95% CI]), albeit with a moderately high posterior predictive error rate of 0.513. As all parameter estimations were right‐skewed, we considered the mode as the best measure of centrality for each scenario. Our analysis indicates that the ancestral population (*N*_*anc*_ = 27.8) encountered a population bottleneck very recently (*t*_*bot*_ = 12.5 generations ago), with the effective population size shrinking to <10 (*N*_*bot*_ = 2) before recovery (*N*_*rec*_ **= 9.94). The DIYABC current effective population size estimate is in good agreement with our estimates using the molecular coancestry approach in NeEstimator (*N*_*e*_ = 4.6 [4.1, 5.2; 95% CI]).

### 3.4 Dispersal

Regarded as a single population, the rock pigeons in Singapore have a slightly higher *H*_e_ (0.159 ± 0.002) than *H*_o_ (0.151 ± 0.002), and a positive inbreeding coefficient (*F*_IS_ = 0.043). Spatial autocorrelations on three different datasets (among males, among females, and among all individuals) gave similar genetic patch sizes (Supporting Information Figure S3) indicating no sex‐biased dispersal. In spatial autocorrelation, intercepts with zero were at 6.412 km, 7.765 km, and 9.407 km (male, female, and total, respectively), while intercepts with the upper boundary (U) of the null model were at approximately 3 km.

**Figure 4:**
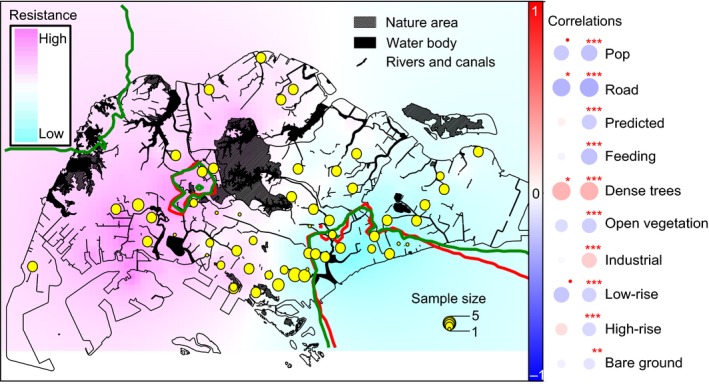
Resistance to rock pigeon dispersal and its correlation with environmental covariates across Singapore. Darker pink‐colored area indicates higher resistance for dispersal, whereas darker blue‐colored area indicates lower resistance. Red contour line encloses areas that have sufficient statistical power, and dark green contour line encloses areas that have sufficient statistical significance. Two columns of circles indicate correlations within the contour line area (left) and overall Singapore (right). Abbreviation of environmental covariates: Pop: human population density; Road: road density; Predicted: predicted pigeon density; Feeding: intensity of intentional feeding incidents. For p‐values of correlations, ● 0.05 < *p* < 0.1, * 0.01 < *p* < 0.05, ** 0.001 < *p* < 0.01, *** 0 < *p* < 0.001

Result of the spatially explicit and individual‐based analysis, DResD, displayed a distribution of positive residuals, indicating resistance to dispersal, in the west of Singapore, with a distribution of negative residuals in the east (Figure 4). Two areas of residual distribution, both of which significantly deviated from the randomized residual distribution, were detected: One in the central west of Singapore constituted a dispersal barrier characterized by positive residuals, and one along the southeast coast of Singapore constituted a dispersal corridor characterized by negative residuals (Figure 4). With respect to areas in which residuals were statistically significant, the presence of dense trees was positively correlated to dispersal resistance, whereas a high road density was negatively correlated to dispersal resistance; all other environmental covariates displayed no significant (*p *>* *0.05) correlations (Figure 4, Supporting Information Figure S4a). Overall, resistance to dispersal was positively correlated with the presence of dense trees and industrial landscapes and negatively correlated with other environmental covariates (Figure 4, Supporting Information Figure S4b).

## 4 DISCUSSION

Our ecological and genomic analyses indicate that rock pigeons in Singapore constitute a single population (Figure 3, Supporting Information Figure S2) of approximately 190,000 individuals. A relatively small genetic patch size (Supporting Information Figure S3) suggests limited dispersal among different pigeon colonies. Human activities as well as man‐made structures play a significant role in rock pigeons’ distribution and dispersal, which can best be predicted by their level of exposure to mercy feeding by humans and by the kind of urban landscape they inhabit (Figure 2). Therefore, higher densities of pigeons are usually found near areas with a higher incidence of mercy feeding, especially in high‐rise areas. In addition, areas with dense trees effectively deter the dispersal of rock pigeons, whereas a high road density promotes their dispersal (Figure 4, Supporting Information Figure S4).

### 4.1 Origin and a possible invasion scenario

The introduction of rock pigeons to Singapore is relatively recent. The earliest record of rock pigeons in Singapore relates to a group kept in the Singapore Botanical Gardens for exhibition (Ridley, 1906). Only a few hundred additional rock pigeons were present in a limited range within the city center half a century ago (Ward, 1968). Although it is unclear whether these two groups of rock pigeons were related, the former group was under management, whereas the latter was characterized by a feral lifestyle typical of many urban pigeon populations nowadays. According to our demographic history analyses using ABC, the most likely evolutionary scenario is a relatively recent introduction followed by population expansion and a small estimated present‐day effective population size (<10 individuals). We traced the bottleneck to around 12 generations ago, implying an introduction timing of 12–84 years ago, considering that generation time in rock pigeons varies from 1 to 7 years (Johnston & Janiga, 1995). According to our genetic clustering results (Figure 3, Supporting Information Figure S2), all rock pigeons in Singapore are likely descendants of those individuals documented in 1968 or a similar group of individuals. Moreover, individuals present in 1968, whether from a single or multiple origins, must have existed as a relatively small and homogeneous population for a few generations before expanding to the rest of Singapore. According to our genetic patch size estimates (Supporting Information Figure S3), Singapore's present rock pigeon population is locally clustered and unlikely to be panmictic. Instead, the single population is likely the consequence of a lack of diversification after rapid growth (from a dozen to ~190,000 individuals within ~50 years) and expansion from a small and genetically uniform group of individuals. In addition, lack of genetic diversity across the entire island of Singapore suggests limited immigration beyond the Johor Straits from Malaysia, which also corresponds to the observation that pigeons are not likely to cross large bodies of water (Jerolmack, 2013).

### 4.2 Abundance and distribution

Our study provides a first total estimate of rock pigeons in Singapore of 189,943 individuals (3.59 individuals per hectare). There have been multiple studies of rock pigeon density around the world's cities: 0.11 individuals per hectare in Islamabad and Rawalpindi, Pakistan (Ali, Rakha, Hussain, Nadeem, & Rafique, 2013); 0.1 to 5.6 individuals per hectare, depending on human population density, in Amsterdam, Holland (Buijs & Van Wijnen, 2001); 4.5 individuals (in summer) to 6.8 individuals (in winter) in Wellington, New Zealand (Ryan, 2011); 9.4 individuals in Barcelona, Spain (Sol & Senar, 1992); 4.34 individuals (in farmland) to 20.83 individuals (in the city center) per hectare in Milan, Italy (Sacchi et al., 2002); 13.88 individuals (during January and February) to 24.71 individuals (during November) per hectare in Pisa, Italy (Giunchi, Gaggini, & Baldaccini, 2007). In comparison, the overall average density of rock pigeons in Singapore was relatively low, although the maximum rock pigeon density of a specific area reached 43.41 individuals per hectare. The great variability in rock pigeon density estimates across the world's cities is at least partly attributable to how cities are delineated in each study. In our case, we are including the whole main island of Singapore (719.1 km^2^), which is mostly urbanized but additionally comprises smaller areas of secondary forest, woodland, agricultural areas, and wasteland. Our inner city estimates of >40 individuals per hectare would appear to put Singapore at the higher end of urban pigeon densities.

The unevenness of rock pigeon distribution across Singapore (Figure 1) is mainly associated with differences across the urban landscape and differences in exposure to mercy feeding by humans, as indicated in the L‐FL model (Figure 2).

Our results agree with Przybylska et al. (2012) who also found the presence of tall buildings as a significant explanatory predictor of pigeon density. A high‐rise landscape is favorable as it superficially resembles rock pigeons’ natural habitats of rock faces, caves, and cliffs (Gibbs, Barnes, & Cox, 2001). Tall buildings allow pigeons to roost and nest at their preferential heights (>20 m) above ground to avoid predation and other disturbances (Haag‐Wackernagel & Geigenfeind, 2008; Harris, De Crom, Labuschagne, & Wilson, 2016; Pikula, Beklová, & Kubik, 1982). Therefore, high‐rise buildings are capable of providing preferable nesting conditions, accommodating fairly large quantities of rock pigeons. These factors may account for the much higher pigeon density in high‐rise landscapes as compared to other areas with the same volume of human feeding, as predicted by the L‐FL model (Figure 2).

Food resources consistently correlate strongly with urban bird population density (Fuller, Warren, Armsworth, Barbosa, & Gaston, 2008; Marzluff, Bowman, & Donnelly, 2001). Therefore, if correctly accounted for, food resources should often be predictive of pigeon density. The failure of previous studies to link food resources with rock pigeon density (Przybylska et al., 2012) may be related to misinterpreting rock pigeons’ main food sources as coming from unintentional feeding (litter bins, grocery stores, and fast‐food restaurants) as opposed to intentional mercy feeding. Previous observations (Sol, Santos, Garcia, & Cuadrado, 1998) have indicated that intentional feeding attracts far more individuals than unintentionally generated food waste does.

### 4.3 Population dynamics

Previous studies suggested a lack of genetic exchange (Jacob et al., 2015) and a tendency of mating with relatives (Jacob et al., 2016) among rock pigeons in Paris. Our estimates of positive *F*_IS_ and a relatively small genetic patch size support these characteristics in Singapore's rock pigeon population. The lack of dispersal, indicated by a small genetic patch size in spatial autocorrelation (Supporting Information Figure S3), is not in disagreement with the lack of a clear spatial genetic pattern observed in the PCA (Figure 3), because the PCA reflects all pairwise comparisons island‐wide, whereas only a small proportion of individual pairs (within 3 km) account for the spatial genetic pattern in spatial autocorrelation. Moreover, rock pigeons were relatively less dispersed, and had a relatively smaller genetic patch size when compared with another common human commensal bird species, the Javan myna (*Acridotheres javanicus*), in Singapore (Low et al., 2018).

Local food sufficiency may lead to limited dispersal and inbreeding of rock pigeons. For rock pigeons, the foraging range normally defines the breeding range as the courtship rituals often take place at the feeding sites (Johnston & Janiga, 1995). Our estimate of a ~3 km genetic patch size (Supporting Information Figure S3) corresponds to field observations of a maximum 5.29 km foraging range, with 92.5% of individuals foraging across less than a 2 km range (Rose, Nagel, et al., 2006). In a typical manner, female dispersal exceeds male dispersal, as characterized by the reproductive behavior of male pigeons attracting females to the male's colony after the female's acceptance of male's courtship (Hetmański, 2007; Johnston & Janiga, 1995). Our spatial autocorrelation result of a ~3 km genetic patch with non‐sex‐biased dispersal suggests that male pigeons tend to attract females from the same colony due to their limited foraging range. Although mercy feeding leads to an increase in pigeon abundance, it does not necessarily influence the dispersal range of a rock pigeon (Rose, Haag‐Wackernagel, & Nagel, 2006). Therefore, mercy feeding provides sufficient food for local pigeon population growth, but does not necessarily recruit individuals from distant places (Figure 4; Supporting Information Figure S4a).

We detected that resistance to dispersal was higher in the north and west, as compared to the south and east of Singapore (Figure 4). This was probably a consequence of landscape differences: in the north and west, areas of high‐rise landscape are fragmented, as they are segregated by dense trees or industrial landscape. In contrast, areas of high‐rise landscape are continuous or connected through low‐rise landscape in the south and east (Supporting Information Figure S1b). The statistically significant dispersal barrier detected to the west of the forested central catchment may suggest that northern and western rock pigeons are genetically more isolated than other Singaporean populations (Figure 4). As for the significant dispersal corridor detected along the east coast, it indicates relatively frequent dispersal among the densest colonies in the east, center, and south (Figure 1), in agreement with the high road density in that area, suggesting that dispersal may be the consequence of exploring new feeding sites by unpaired young individuals. These young individuals are less competitive than adults on local food resources (Sol, Santos, & Cuadrado, 2000; Sol et al., 1998) and therefore may move along road corridors to navigate to distant feeding sites (Dell'Ariccia, Dell'Omo, Wolfer, & Lipp, 2008; Lipp et al., 2004). Our estimates of a higher pigeon density in the east than the west (Figure 1) may additionally explain the higher dispersal rates in the east than in the west.

### 4.4 Implications and limitations

Our study suggests a strong impact of mercy feeding activities and urban landscape features on the distribution and dispersal of rock pigeons across Singapore. Manipulating these two factors may be key in maintaining a balanced rock pigeon population, which incurs a minimum impact on the well‐being of the public. Given the relatively low levels of pigeon dispersal demonstrated by our population genomic analyses, we propose an implementation of rigorous management methods at local hotspots with high ecological and social risks: First, in light of the significant correlation between mercy feeding and rock pigeon density, educational programs (Haag‐Wackernagel, 1993; Johnston & Janiga, 1995) to reduce intentional feeding may effectively limit the growth of local rock pigeon colonies. Second, given the high pigeon densities in high‐rise landscapes, methods of modifying high‐rise landscapes, for example, through the introduction of pigeon deterrents, may help reduce their carrying capacity (Giunchi, Albores‐Barajas, Baldaccini, Vanni, & Soldatini, 2012; Haag‐Wackernagel & Geigenfeind, 2008). At last, planting more trees, especially in areas of high road density, may help strengthen the effect of local population control by curbing the influx of rock pigeons from neighboring colonies.

While we provide comprehensive information on the dispersal patterns of rock pigeons across Singapore, the geographical origin of Singaporean rock pigeons is still unknown. To answer the question, future studies may consider expanding sampling effort to compare Singaporean individuals to urban pigeon populations from other Asian cities and worldwide. Moreover, this study concentrated on four environmental covariates. Untested covariates, for example, age of buildings (Sacchi et al., 2002), may provide additional clues as to the distribution and dispersal of rock pigeons in Singapore: Places we identified as a corridor are congruent with the relatively old and cultural part of Singapore. In the future, as computational power continues to improve, more environmental covariates can be explored with sophisticated models to produce more accurate and highly resolved maps of rock pigeon distribution and dispersal. At last, our study predominantly used genetically neutral markers. Future studies can expand to protein‐coding genes or mRNA to discover the long‐term human influence on rock pigeon evolution (Alberti, 2015).

## CONFLICT OF INTEREST

None declared.

## DATA ARCHIVING STATEMENT

Data available from the Dryad Digital Repository: https://doi.org/10.5061/dryad.gp864h7
