# Supplementary figures and images for: Human activities and landscape features interact to closely define the distribution and dispersal of an urban commensal
Source: Evol Appl. 2018 Jul 21;11(9):1598–608. doi: 10.1111/eva.12650 (PMC6183452; doi:10.1111/eva.12650)

(a)

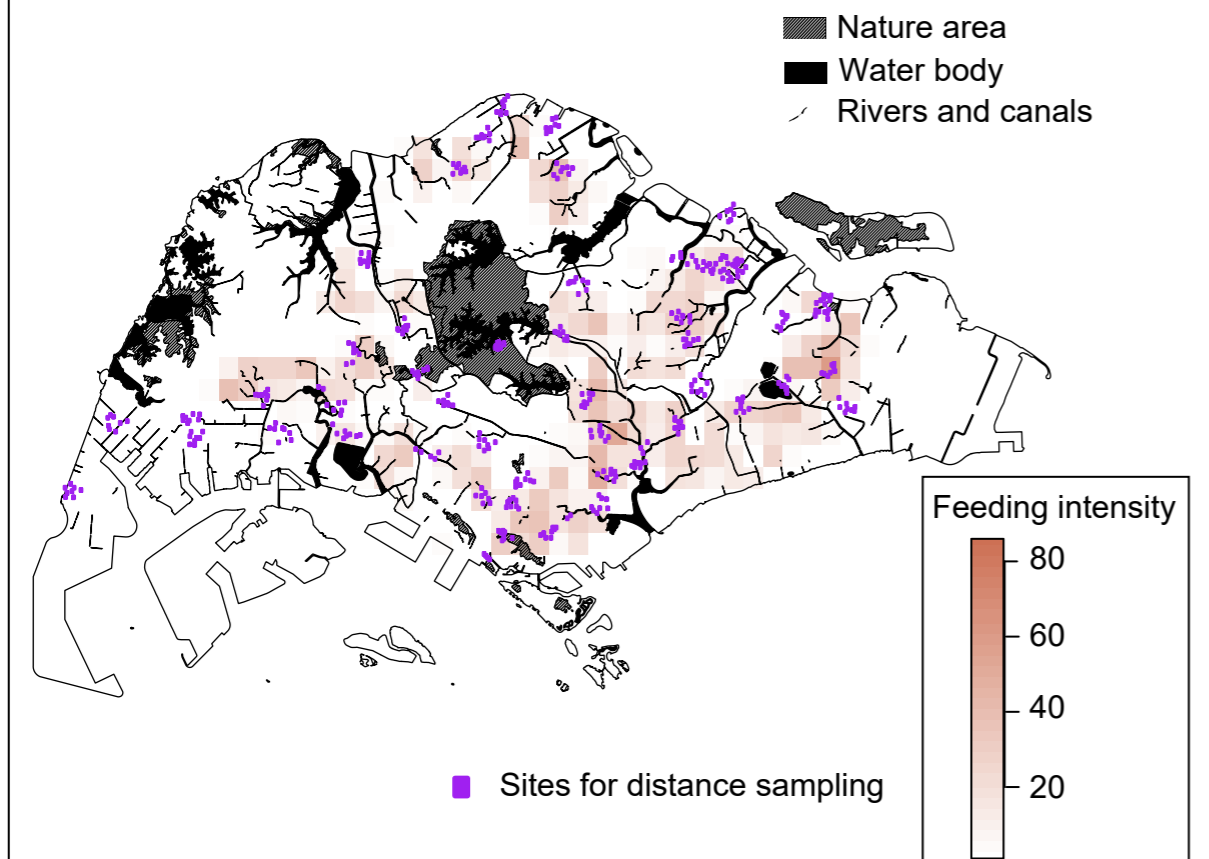

(b)

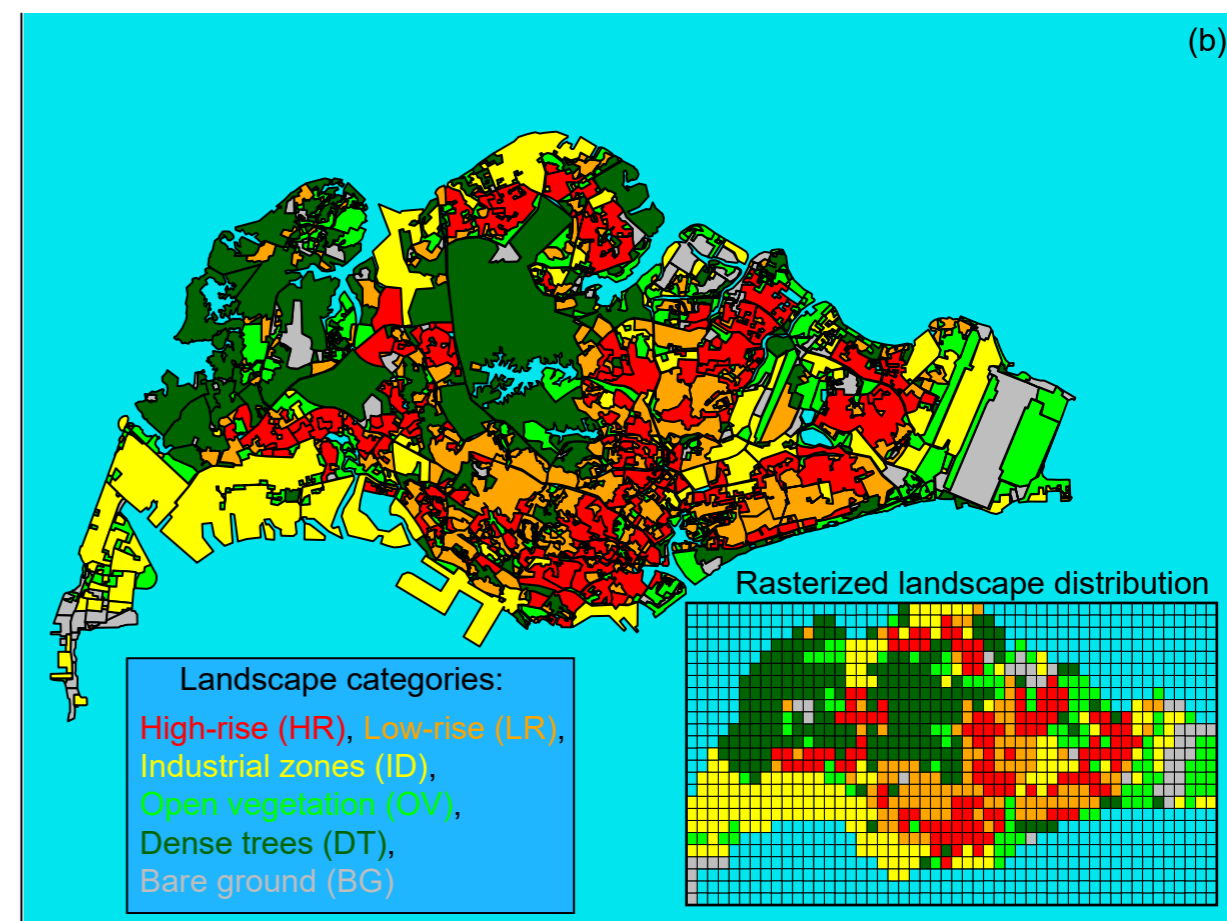

(c)

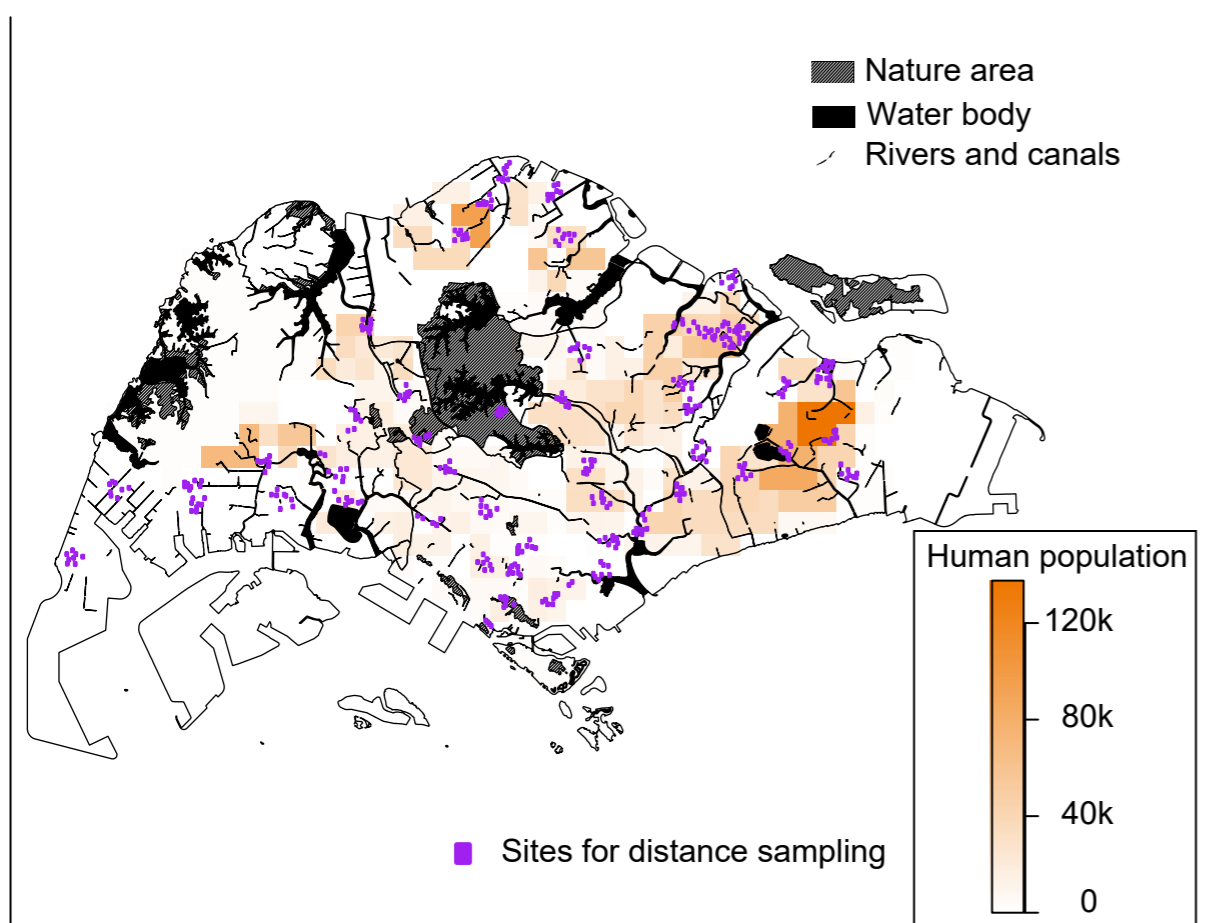

(d)

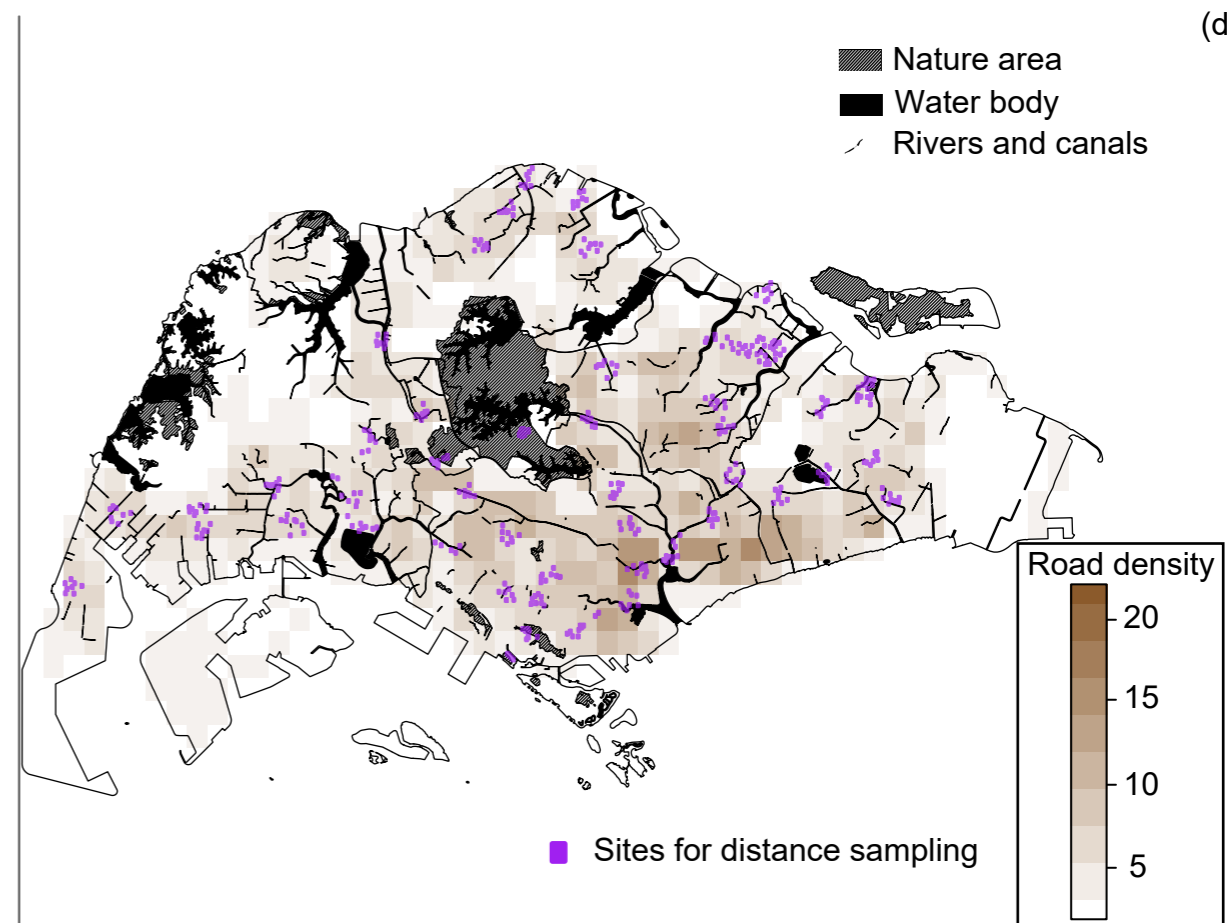

- Nature area
- Water body
- Rivers and canals

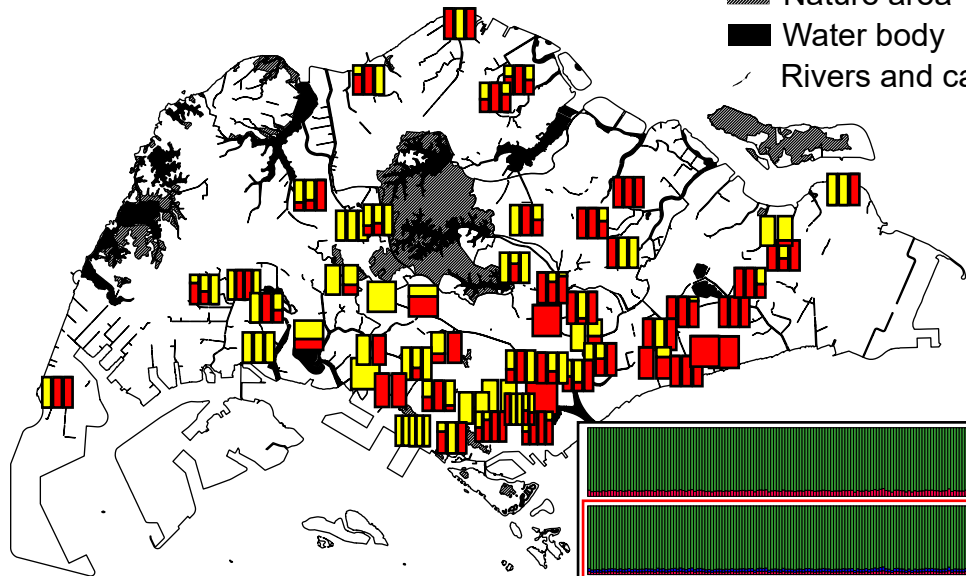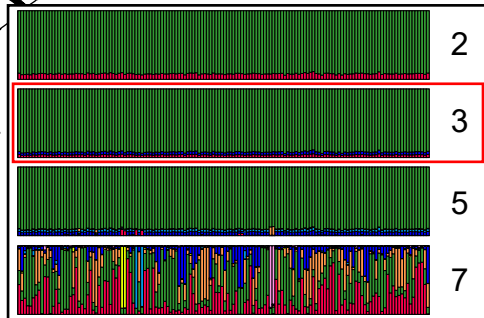

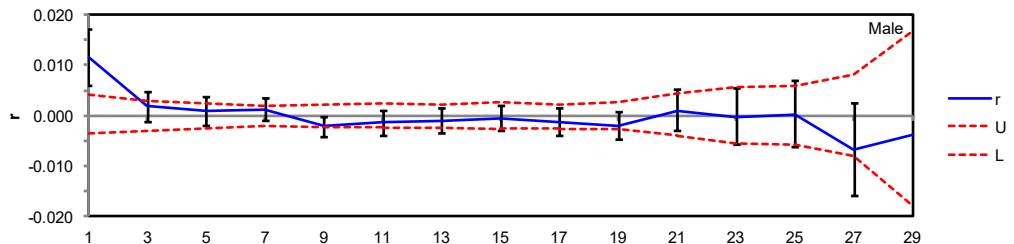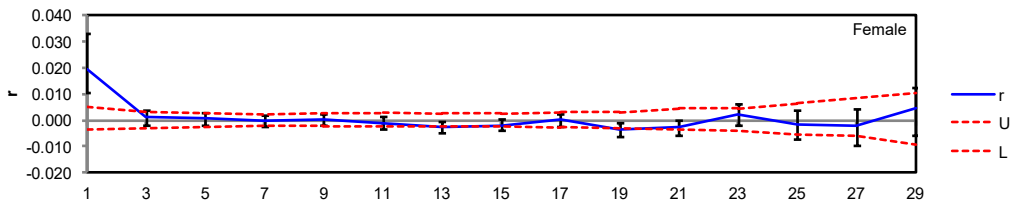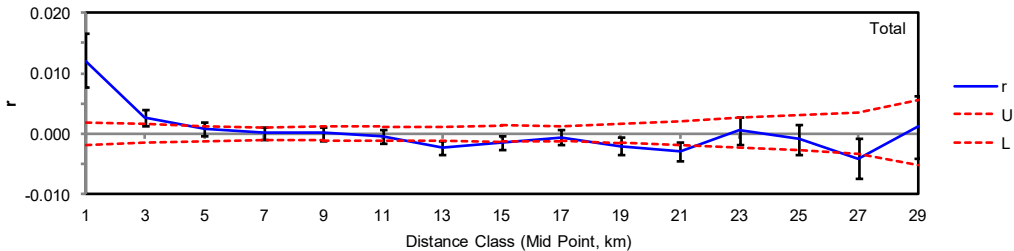

Supplement: Supplementary file 1 [file EVA-11-1598-s001.pdf]
