# Supplementary material for: Human activities and landscape features interact to closely define the distribution and dispersal of an urban commensal
Source: Evol Appl. 2018 Jul 21;11(9):1598–608. doi: 10.1111/eva.12650 (PMC6183452; doi:10.1111/eva.12650)
Supplement: Supplementary file 2 [file EVA-11-1598-s002.docx]

**Table S1. Models tested in this study**.

| Name | Detection probability (*p*) | Abundance (λ) | Parameters | AIC | ΔAIC | Relative likelihood | *wt* |
| --- | --- | --- | --- | --- | --- | --- | --- |
|  |  |  |  |  |  |  |  |
|  |  |  |  |  |  |  |  |
| L-FL | Landscape | Landscape + feeding intensity | 12 | 2300.77 | 0 | 1 | 0.45 |
| LR-FL | Landscape + road density | Landscape + feeding intensity | 13 | 2302.4 | 1.63 | 0.442639327 | 0.20 |
| FL-FL | Landscape + feeding intensity | Landscape + feeding intensity | 13 | 2302.7 | 1.93 | 0.3809832 | 0.17 |
| L-FLR | Landscape | Feeding density + landscape + road density | 13 | 2302.77 | 2 | 0.367879441 | 0.17 |
| FL-F | Landscape + feeding intensity | Feeding density | 9 | 2308.85 | 8.08 | 0.017597472 | 0.01 |
| FL-FR | Landscape + feeding intensity | Feeding density + road density | 10 | 2310.9 | 10.13 | 0.006313911 | 2.85E-3 |
| L-F | Landscape | Feeding density | 8 | 2314.63 | 13.86 | 0.000978001 | 4.41E-4 |
| LR-FR | Landscape + road density | Feeding density + road density | 10 | 2315.69 | 14.92 | 0.000575656 | 2.6E-4 |
| LR-F | Landscape + road density | Feeding density | 9 | 2316.22 | 15.45 | 0.000441647 | 1.99E-4 |
| L-FR | Landscape | Feeding density + road density | 9 | 2316.62 | 15.85 | 0.00036159 | 1.63E-4 |
| F-L | Feeding density | Landscape | 8 | 2357.41 | 56.64 | 5.02E-13 | 2.26E-13 |
| F-F | Feeding density | Feeding density | 5 | 2363.69 | 62.92 | 2.17E-14 | 9.80E-15 |
| L-L | Landscape | Landscape | 11 | 2369.23 | 68.46 | 1.36E-15 | 6.14E-16 |
| N-F | Null | Feeding density | 4 | 2373.91 | 73.14 | 1.31E-16 | 5.91E-17 |
| N-L | Null | Landscape | 7 | 2385.71 | 84.94 | 3.59E-19 | 1.62E-19 |
| R-F | Road density | Feeding density | 8 | 2386.04 | 85.27 | 3.05E-19 | 1.37E-19 |
| F-R | Feeding density | Road density | 8 | 2408.73 | 107.96 | 3.60E-24 | 1.63E-24 |
| L-N | Landscape | Null | 7 | 2408.75 | 107.98 | 3.57E-24 | 1.61E-24 |
| F-N | Feeding density | Null | 4 | 2422.69 | 121.92 | 3.35E-27 | 1.51E-27 |
| R-N | Road density | Null | 4 | 2479.8 | 179.03 | 1.33E-39 | 6.00E-40 |
| N-R | Null | Road density | 4 | 2480.68 | 179.91 | 8.57E-40 | 3.86E-40 |
| R-R | Road density | Road density | 5 | 2481.68 | 180.91 | 5.20E-40 | 2.34E-40 |
| N-N | Null | Null | 3 | 2489.75 | 188.98 | 9.19E-42 | 4.15E-42 |
| N-P | Null | Residence population | 4 | 4933.5 | 2632.73 | 0 | 0 |
| F-P | Feeding density | Residence population | 8 | 4941.5 | 2640.73 | 0 | 0 |
| FL-FP | Landscape + feeding intensity | Feeding density + residence population | 10 | 4945.5 | 2644.73 | 0 | 0 |
| LR-FP | Landscape + road density | Feeding density + residence population | 10 | 4945.5 | 2644.73 | 0 | 0 |
| L-FLP | Landscape | Feeding density + landscape + residence population | 13 | 4951.5 | 2650.73 | 0 | 0 |
| L-FLRP | Landscape | Feeding density + landscape + residence population + road density | 14 | 4953.5 | 2652.73 | 0 | 0 |
| LP-FP | Landscape + residence population | Feeding density + residence population | 10 | 4967.82 | 2667.05 | 0 | 0 |
| P-N | Residence population | Null | 4 | 5587.72 | 3286.95 | 0 | 0 |
| P-F | Residence population | Feeding density | 8 | 5595.72 | 3294.95 | 0 | 0 |
| LP-F | Landscape + residence population | Feeding density | 9 | 5597.72 | 3296.95 | 0 | 0 |
| LP-FR | Landscape + residence population | Feeding density + road density | 10 | 5599.72 | 3298.95 | 0 | 0 |
| LP-FL | Landscape + residence population | Landscape + feeding intensity | 13 | 5605.72 | 3304.95 | 0 | 0 |
| P-P | Residence population | Residence population | 4 | 6889.26 | 4588.49 | 0 | 0 |
