# Supplementary material for: Human activities and landscape features interact to closely define the distribution and dispersal of an urban commensal
Source: Evol Appl. 2018 Jul 21;11(9):1598–608. doi: 10.1111/eva.12650 (PMC6183452; doi:10.1111/eva.12650)
Supplement: Supplementary file 3 [file EVA-11-1598-s003.docx]

**Fig. S1. Environmental covariates used in this study.** (a) Feeding intensity (density of intentional feeding incidents); (b) landscape categories; (c) distribution of human residents across Singapore; (d) road density (in km).

**Fig. S2.** **Genetic clustering using maximum likelihood and Bayesian inference methods.** Maximum likelihood clustering using *ADMIXTURE* indicates two genetic clusters mapped according to sampling locations. Bayesian clustering using STRUCTURE is displayed as bar plots at the right bottom, with the most likely scenario (three clusters) highlighted.

**Fig. S3. Spatial autocorrelations of genetic coefficients (r) for male, female and total pigeon individuals across Singapore.** Null model of spatial structure indicated within upper (U) and lower (L) 95% confidence limits.

**Fig. S4. Correlations among dispersal resistance (Residual), predicted pigeon density (Predicted) and environmental covariates.** (a) Correlations within areas of residuals that were characterized by a sufficient statistical significance and power; (b) Correlations across all studied areas in Singapore. Histograms along the diagonal display the data distribution; scatter plots below the diagonal indicate correlation performance; values above the diagonal indicate the coefficient and the significance (*p*-values) of correlations. Abbreviation of environmental covariates: Pop = human population density; Road = road density; Predicted = predicted pigeon density (see Fig. 1); Feeding = density of intentional feeding incidents; DT, OV, ID, LR, HR, BG (see Table 1). For *p*-values of correlations, · 0.05<*p*<0.1, * 0.01<*p*<0.05, ** 0.001<*p*<0.01, *** 0<*p*<0.001.
